# Phytochemical Composition, Antioxidant, and Anticancer Activities of Sidr Honey: In Vitro and In Silico Computational Investigation

**DOI:** 10.3390/life13010035

**Published:** 2022-12-23

**Authors:** Nouha Bouali, Walid Sabri Hamadou, Riadh Badraoui, Ramzi Hadj Lajimi, Assia Hamdi, Mousa Alreshidi, Mohd Adnan, Zohra Soua, Arif Jamal Siddiqui, Emira Noumi, Mejdi Snoussi

**Affiliations:** 1 Department of Biology, College of Science, University of Hail, P.O. Box 2440, Hail 2440, Saudi Arabia; 2 Research Unit: Molecular Biology of Leukemia and Lymphoma, Department of Biochemistry, University of Medecine of Sousse, Sousse 4002, Tunisia; 3 Section of Histology—Cytology, University of Medicine of Tunis, University of Tunis El Manar, La Rabta 1007, Road Djebal Lakhdhar, Tunis 1007, Tunisia; 4 Department of Histo-Embryology and Cytogenetics, Medicine Faculty of Sfax, University of Sfax, Road of Majida Boulia, Sfax 3029, Tunisia; 5 Department of Chemistry, College of Science, University of Hail, P.O. Box 2440, Hail 2440, Saudi Arabia; 6 Laboratory of Water, Membranes and Environmental Biotechnologies, Center of Research and Water Technologies, P.O. Box 273, Soliman 8020, Tunisia; 7 Laboratory of Galenic and Pharmacological Chemical Development of Drugs, University of Pharmacy, Monastir 5000, Tunisia; 8 Laboratory of Genetics, Biodiversity and Valorisation of Bioressources, High Institute of Biotechnology University of Monastir, Monastir 5000, Tunisia

**Keywords:** anticancer, antioxidant, computational analysis, HR-LCMS, *Ziziphus* honey

## Abstract

Cancer is one of the major causes of death worldwide. The repercussions of conventional therapeutic approaches present a challenge in the delivery of new effective treatments. Thus, more attention is being awarded to natural products, mainly honey. Honey could be the basis for the development of new therapies for cancer patients. The aim of this study is to assess the phytochemical profiling, antioxidant, drug-likeness properties, and anticancer activity of *Ziziphus* honey (ZH) derived from the Hail region of Saudi Arabia. The phytochemical profiling using high resolution-liquid chromatography mass spectrometry (HR-LCMS) revealed 10 compounds belonging to several familial classes and one tripeptide. Potential antioxidant activity was noted as assessed by DPPH (IC_50_ 0.670 mg/mL), ABTS (IC_50_ 3.554 mg/mL), and β-carotene (IC_50_ > 5 mg/mL). The ZH exerted a notable cytotoxic effect in a dose-dependent manner against three cancer cell lines: lung (A549), breast (MCF-7), and colon (HCT-116), with respective IC_50_ values of 5.203%, 6.02%, and 7.257%. The drug-likeness investigation unveiled that most of the identified compounds meet Lipinski’s rule. The molecular docking analysis revealed interesting antioxidant and anticancer activities for most targeted proteins and supported the in vitro findings. The Miraxanthin-III compound exhibited the most stabilized interaction. This study provides deeper insights on ZH as prominent source of bioactive compounds with potent antioxidant and anticancer effects.

## 1. Introduction

Cancer represents a heavy burden on healthcare systems and is a major public concern globally. According to the International Agency for Research on Cancer (IARC), the number of diagnosed cases of cancer is increasing. Nearly 19.3 million new cases were recorded in 2020. Breast, lung, and colorectal cancer are the main types of cancer detected in both males and females, with respective rates of 11.7%, 11.4%, and 10% [1]. The number of new diagnostic cases of cancer may rise to 32.2 million in 2040 [2].

Despite thorough research targeting the prevention of cancer and providing efficient, safe treatments, the disease continues to gain range and meets challenges to overcome this ubiquitous issue. The conventional approaches of treating cancer by chemotherapy, radiation therapy, and surgery often have severe side effects on patients and are very painful. Furthermore, some malignancies may display drug resistance leading to poor prognosis and poor survival among patients. This is why complementary and alternative medicine could be a valuable substitute, offering new potential bioactive molecules with therapeutic effects. Among these natural products is honey, which is often considered one of the best sources of potent phytochemicals exhibiting interesting chemopreventive and chemotherapeutic effects against cancers [3].

Honey has been used in folk medicine and has been known for its healing properties for many years. Prominent therapeutic results have been reported to treat infectious and inflammatory diseases. Recently there has been an increased interest in honey phytochemicals and their pharmacological application in cancers. Several results showed prominent anticancer activity displayed by various honey types [4]. Honey content may vary according to floral origin, the geographical area considering the same floral origin, season, altitude, and species of honeybees [4,5,6]. Monofloral honey derived from the nectar of one plant species has a high virtue compared with polyfloral honey [7]. Saudi Arabia is among the geographic regions rich in valuable monofloral honey, such us *ziziphus* honey (*Ziziphus* spp.) (ZH) is also known as Sidr honey [8]. ZH is derived from three main plant species, namely *Ziziphus* nummularia, *Z. mucronata*, and *Z. spina*-christi [9].

*Ziziphus* spp. is considered a medicinal plant for its therapeutic virtue [10,11,12]. Consequently, valuable phytochemicals or their metabolites could be provided by ZH. Several studies reported interesting antioxidant, anti-inflammatory, and antitumor effects related to the bioactive phytochemical content in ZH. The chemopreventive effect is due mainly to antioxidant bioactive molecules, mainly polyphenols and flavonoids, which may perform scavenging activity against free radicals preventing tumorigenesis processes [13]. The anticancer potential exhibited by ZH is associated with polyphenolic profiles, such as carvacrol and its isomer thymol, found in *Ziziphus* Spina-Christi honey [14,15]. Both of them include compounds which may induce cytotoxicity, cell cycle arrest, apoptosis, antimetastatic activity, and may lead to inhibition of several signaling pathways and antiproliferative effects [15].

A previous in vivo study highlighted that ZH can display preventive effects against gastric ulceration in rats through antioxidant and anti-apoptotic effects [16]. In addition, it has been proven that honey could be a valuable chemoprotectant or adjuvant during cancer treatment [17]. An in vitro study showed that ZH might improve breast cancer treatment by modulating the expression of genes involved in metastasis in triple-negative breast cancer cell lines [18]. A clinical trial disclosed also that ZH could reduce significantly the severity of radiotherapy-induced mucositis in patients with head and neck cancers [18]. All these finding highlight the chemopreventive and chemotherapeutic effects displayed by ZH.

In a previous study, we studied *Acacia* honey derived from the Hail region. Interesting results have been reported highlighting the potential use of this honey as a source of bioactive compounds with significant antimicrobial, antioxidant, and anticancer activities, which could imply further pharmacological application. Thus, more honey subtypes should be investigated [19]. This study seeks to assess the biological activities of ZH obtained from the Hail region for the first time. The HR-LCMS method was used to investigate the phytochemical profile of ZH from the Hail geographical floral region. This methodology was used to assess the total phenols, flavonoids, and tannins, and to measure the antioxidant potential. This study will also estimate the anticancer activity of ZH against lung (A549), breast (MCF-7), and colon (HCT-116) cancer cell lines. Finally, a computational study was conducted in order to estimate the potential affinity based on conformation and complementarity between identified compounds and the active binding site of a targeted protein to explore the anticancer and antioxidant effects of ZH [19]. The drug-likeness properties were also assessed.

## 2. Materials and Methods

### 2.1. Honey Sampling

The ZH was produced from apiaries in the Hail region and was conserved at 4 °C in dark, dry, and sterile conditions. The monofloral origin was verified by microscopic observation of the honey to check for predominant pollens [20].

### 2.2. Phytochemical Screening of ZH

#### 2.2.1. Polyphenol Evaluation

The total phenolic compounds in the ZH sample were measured using the Folin-Ciocalteu protocol [21]. A total of 1000 μL of ZH was added to 5 mL of Folin-Ciocalteu reagent (mixed with distilled water 1:10 *v*/*v*) and 4 mL (75 g/L) of sodium carbonate. The samples were vortexed for 15 s then left to stand for 30 min at 40 °C for staining. The absorbance was measured at a wavelength of 765 nm using the Thermo Scientific Spectrascan UV 2700 dual-beam spectrophotometer. The total phenolic content of ZH was expressed as mg/g n-propyl gallate equivalent (mg GAE/g).

#### 2.2.2. Flavonoid Estimation

A total of 2 mL of distilled water and a NaNO2 (0.15 mL; 5%) solution were added to 0.5 mL of ZH then incubated at room temperature for 6 min. A solution of AlCl3 (0.15 mL, 1.1%) was added and allowed to stand for 6 minutes. Afterwards, 2 mL of NaOH (4%) then 5 mL of distilled water were added to the previous mixture. The mixture was allowed to stand for 15 min, and the color intensity was measured at 510 nm. The total phenolic content was expressed in mg of quercetin equivalent (QC) per gram [22].

#### 2.2.3. Determination of Total Tannins

A total of 50 μL of ZH was diluted in 1.5 mL of 4% vanillin then added to 750 μL of concentrated HCl. After vigorous stirring, the mixture was incubated for 20 min at room temperature then the absorbance was measured at 500 nm. The total tannin content was expressed in tannic acid equivalent (TAE) [23].

### 2.3. Antioxidant Activity

The antioxidant activity of ZH was investigated using 3 different approaches, based on electron or hydrogen transfer reactions, to assess scavenging activity: DPPH radical scavenging activity, ABTS radical scavenging activity, and β-carotene-linoleic acid bleaching (BCB) assay [24,25]. The butylated hydroxytoluene (BHT) and ascorbic acid (AA) were used as positive antioxidant sample controls.

#### 2.3.1. DPPH Radical Scavenging Activity

The DPPH (2,2-diphenyl-1-picryl-hydrazyl-hydrate) (Sigma-Aldrich, Milano, Italy) free radical scavenging activities of the ZH samples were determined using an antioxidant assay based on the electron transfer reaction. Several ZH dilutions were incubated with DPPH for 30 min at room temperature. The variation in the color of DPPH was assessed based on spectrophotometric analysis at 515 nm. We used the ascorbic acid as the standard control. The antioxidant activity was calculated using the following formula and expressed as IC_50_ (mg/mL):

[DPPH scavenging activity (%) = (A_0_ − A_1_)/A_0_ × 100];

where A_0_ denotes the absorbance of the control and A_1_ denotes the absorbance of the sample.

#### 2.3.2. ABTS Radical Scavenging Activity

To assess the free radical scavenging activity of ZH, we used ABTS (2, 2-azino-bis (3-ethylbenzothiazoline-6-sulfonic acid); Sigma-Aldrich, Milano, Italy). The ABTS+ was generated by reacting 7 mM ABTS with 2.45 mM K2S2O8 at room temperature under dark conditions. Then, serval ZH dilutions were mixed with 900 μL of the prepared ABTS+ solution and incubated for 30 min. ABTS scavenging activity was calculated using the following formula and expressed as IC_50_ (mg/mL):

[ABTS scavenging activity (%) = (A_0_ − A_1_)/A_0_ × 100];

where A_0_ denotes the absorbance of the control and A_1_ denotes the absorbance of the sample.

#### 2.3.3. β-Carotene/Linoleic Acid Method

The β-carotene method was performed as described by Ikram et al. [26]. An emulsion containing 5 mg β-carotene, 0.30 mL linoleic acid, and 1.5 mL Tween 40 in 650 mL distilled water was prepared. A total of 25 mL from the prepared emulsion was added to 1 mL of ZH then incubated in bath water at 50 °C in the dark. Absorbance of β-carotene was assessed based on spectrophotometric analysis at 470 nm (t = 0 min) and after 2 h of incubation (t = 120 min). The AA and BHT were used as the standard for comparison. The antioxidant activity of ZH was estimated using the following equation: and expressed as IC_50_ (mg/mL):

[PI% = ( A-β-carotene T_120_/A-β-carotene t0) × 100];

### 2.4. MTT Assay

The anticancer activity of ZH was carried out on 3 human cancer cell lines, including lung (A549), breast (MCF-7), and colon (HCT-116) [27]. The National Centre for Cell Science (NCCS) in Pune, India, provided cell lines for use in this study. Doxorubicin (Sigma, Chennai, India) was used as a reference drug. Cells were firstly grown in (Dulbecco’s Modified Eagle Medium) DMEM (MP Biomedicals, Eschwege, Germany) and 10,000 U/mL penicillin and 5 mg/mL streptomycin antibiotic solution (Hi-Media, Mumbai, India) supplemented with 10% fetal bovine serum (FBS) (MP Biomedicals, Germany), then incubated at 37 °C, 5% CO_2_, and 80% humidity. After the required growth of cells was obtained, cells were treated in triplicate for 24 h with variable concentrations of ZH diluted in complete media at 2%, 4%, 6%, 8%, and 10%, then washed with phosphate buffered saline (PBS) solution. The cultured cells were treated for 4 h with the prepared MTT (3-(4,5-dimethylthiazolyl-2)-2,5 diphenyl tetrazolium bromide) (MP Biomedicals, Germany) solution (5 mg/mL). The supernatant was removed and 100 μL DMSO (Merck, Darmstadt, Germany) was added to each well. Cell viability was determined by measuring the absorbance at 570 nm (ELISA reader (EL10 A, Biobase, Jinan, China)) and the percentage of viable cells was estimated in order to determine the 50% cytotoxic concentration IC_50_ values for respective cancer cell lines using the following equation:

[% cell viability = (A_570_ of treated cells/A_570_ of control cells) × 100%];

### 2.5. LCMS Analysis

The phytochemistry of the ZH sample was analyzed using a UHPLC-PDA-Detector Mass Spectrophotometer (HR-LCMS 1290 Infinity UHPLC System), Agilent Technologies®, USA [28]. The liquid chromatographic system consisted of a HiP sampler, binary gradient solvent pump, column compartment, and quadrupole time of flight mass spectrometer (MS Q-TOF) with dual Agilent Jet Stream Electrospray (AJS ES) ion source. A total of 10 μL of the sample was injected into the system, followed by separation in SB-C18 column (2.1 × 50 mm, 1.8 μm particle size). The solvents used were 1% formic acid in deionized water (solvent A) and acetonitrile (solvent B). A flow rate of 0.50 mL/min was used, while MS detection was performed in MS Q-TOF. Compounds were identified via their mass spectra and their unique mass fragmentation patterns. Compound Discoverer 2.1, ChemSpider, and PubChem were used as the main tools for the identification of the phytochemical constituents.

### 2.6. ADMET Analysis

Drug ability and pharmacokinetics were predicted for the different compounds identified in honey based on the ADMET properties (absorption, distribution, metabolism, elimination, and toxicity) using online servers and established commercial packages (http://www.swissadme.ch/) and (http://biosig.unimelb.edu.au/pkcsm/prediction) (accessed on 11 March 2022) as reported in previous studies [29,30,31]. Bioavailability was also studied based on the physicochemical structures of the compounds [29].

### 2.7. Computational Analysis

Three different receptors (human peroxiredoxin 5 (1hd2), cyclin-dependent kinase 2 (CDK-2, 1di8), and rho-associated protein kinase 1 (ROCK 1, 3twj)) were selected. These receptors were targeted to check the potential antioxidant and anticancer effect of the identified compounds. Their crystalized structures were obtained from the RCSB protein data bank. ChemDraw was used to obtain the chemical structures when needed [30,32]. Following the pre-processing of both ligands and receptors, the binding approach was used based on the CHARMM force field as previously described using vina packages [31,33,34]. The binding scores and calculation of embedding distances and bonding networks were studied as previously described [29,30,31]. The reason behind the selection of these receptors is their involvement in antioxidant and anticancer pathways and they are commonly targeted in pharmaceutical and drug design approaches.

### 2.8. Statistical Analysis

For statistical analysis, a significance test was carried out among the treatments by one way ANOVA followed by Tukey’s post hoc test at *p* < 0.05. Statistical analysis was conducted with software GraphPad Prism 5.0.

## 3. Results

### 3.1. Phytochemical Profiling

In order to determine the chemical composition of ZH, high resolution liquid chromatography mass spectrometry (HR-LCMS) was carried out. This technique was performed in the separation and identification of the phytoconstituents based on their retention time, database difference (library), experimental *m*/*z*, MS/MS fragments, metabolite class, and proposed compounds. MS data were provided in negative and positive ionization mode. We revealed 11 compounds, including sugar acids, amino acid derivatives, alkaloids, fatty acids, and one small protein (Supplementary Materials Figure S1). The complete list is summarized in Table 1.

**Figure 1:**
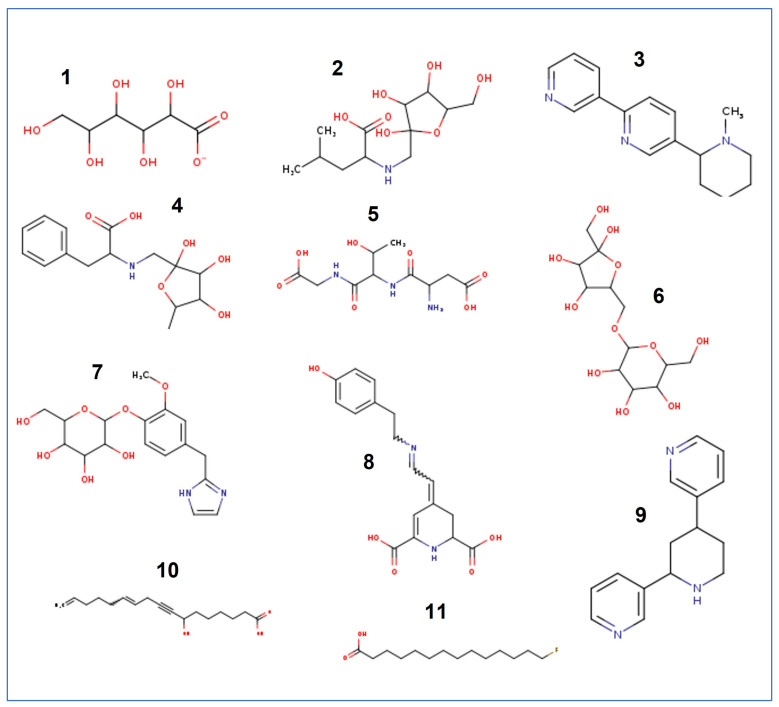
Chemical structure of the identified compounds from *Ziziphus* honey. Names of the compounds are listed in Table 1.

The chemical structures of all identified compounds are listed in Figure 1.

### 3.2. MTT Assay

**Figure 2:**
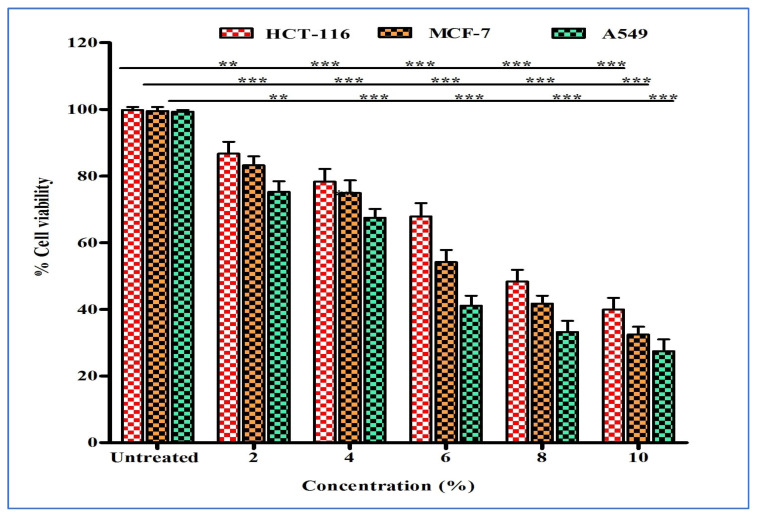
Anticancer activity of honey against A-549, MCF-7, and HCT-116 cancer cells. Error bars indicate SDs (±standard deviation) of three independent experiments. Significance; ** *p* < 0.01, *** *p* < 0.001.

**Figure 3:**
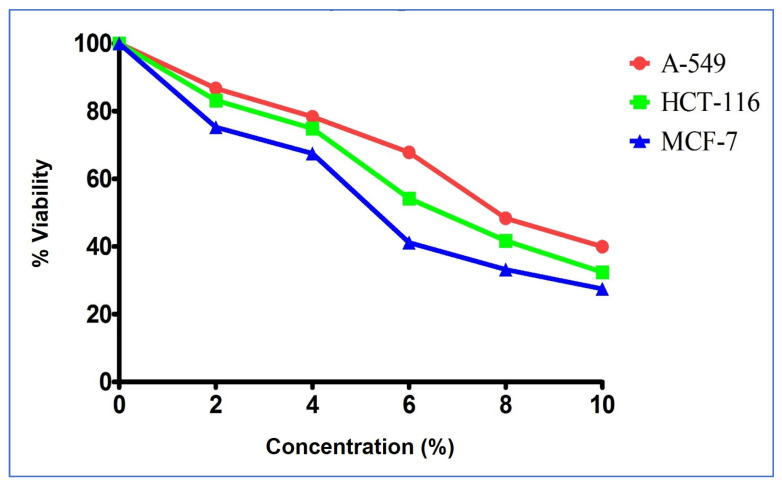
Impact of honey concentration on A-549, MCF-7, and HCT-116 cell line viability.

The cytotoxicity of various concentrations of ZH were assessed after 24 h on three cancer cell lines: breast (MCF-7), lung (A549), and colon (HCT-116). Treatment with ZH significantly decreased cell viability in a dose-dependent manner (Figure 1). The IC_50_ values obtained for breast, colon, and lung cancer were 5.203%, 6.02%, and 7.257%, respectively, while for Doxorubicinin (used as standard) the respective IC_50_ were 3.5 µg/mL, 1.5 µg/mL, and 6.5 µg/mL (Figure 2 and Figure 3).

### 3.3. Anti-Oxidant Potential

The assessment of ZH antioxidant properties was carried out using different approaches. The antioxidant and free radical scavenging activity assessed via DPPH and ABTS methods showed nearly similar values with respective IC_50_ 3.45 mg/mL and 3.554 mg/mL. The β-carotene-linoleic acid bleaching reveled an IC_50_ >5 mg/mL (Table 2). The total phenolic content (TPC), total flavonoids content (TFC), and tannins were illustrated in Table 2.

### 3.4. ADMET Analysis

**Figure 4:**
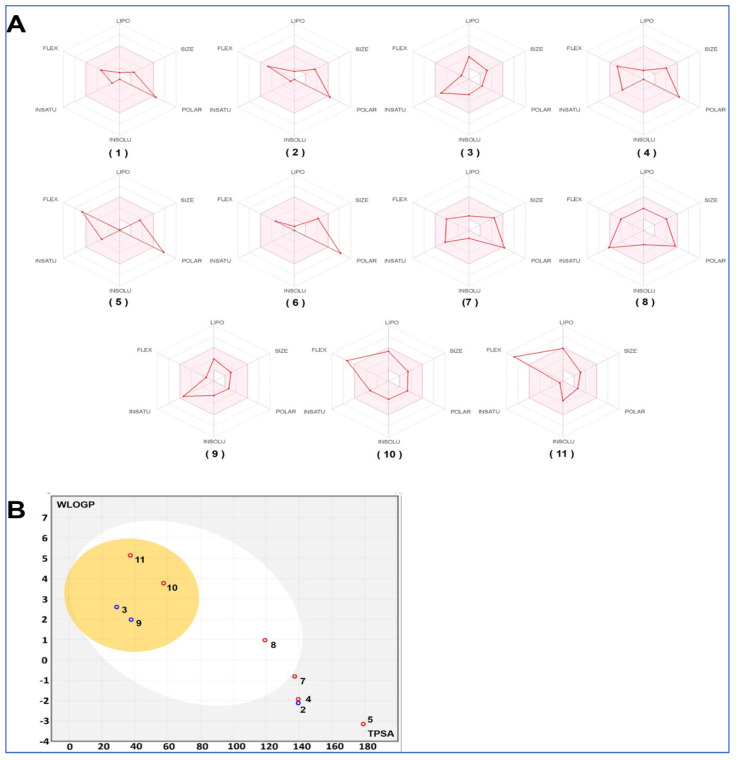
Bioavailability radars (**A**) and boiled-egg model (**B**) of the identified compounds in honey (**1**–**11**).

The investigation of the ADMET properties of the identified compounds from ZH was carried out via the Swiss-model online server. Several measures of physiochemical properties that project the drug-likeness of compounds was carried out. According to Lipinski’s rule of five, a compound of reported biological activity is considered active with good absorption and permeation when the following criteria are fulfilled: the hydrogen-bond acceptors (H-bond acceptors) must be less than 10, hydrogen-bond donors (H-bond donors) less than five, rotatable bonds (RBN) must be 10 or less, a molecular weight not exceeding 500 Da, and a calculated log P (consensus log po/w) less than five. In our analysis, the in silico ADMET study revealed that most of the identified compounds meet Lipinski’s rule with zero alert as shown in Table 3, Figure 4.

### 3.5. Docking Analysis

**Figure 5:**
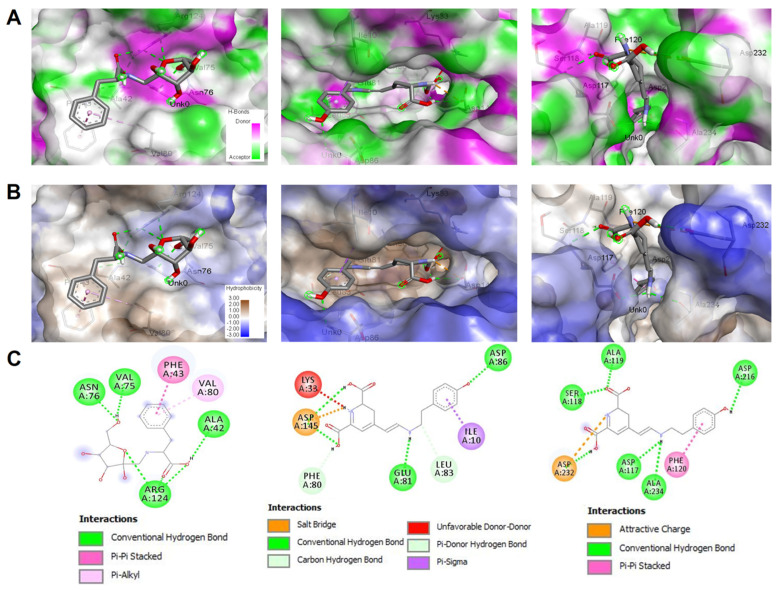
Illustration of 3D H-bonds (**A**), 3D hydrophobic (**B**) and 2D diagrams (**C**) on molecular interactions of the identified compounds in honey and the targeted receptors: 1hd2 (left), adi8 (middle), and 3twj (right), which exhibited the best free-binding energies (−6.6, −8.7 and −8.0 kcal/mol, respectively). The illustrations correspond to the following complexes: 1hd2-compound four, 1di8-compound eight, and 3twj-compound eight.

Table 4 shows that all ZH compounds bound to the three targeted receptors with negative free binding energy, but with different scores. The free binding energy ranged between −4.3 and −8.7 kcal/mol. The best binding score was predicted in the 1di8/Miraxanthin-III (compound No. **8**) complex. Miraxanthin-III was also found to interact with the 3twj receptor with −8.0 kcal/mol. The molecular interactions of this compound with 1DI8 included four conventional H-bonds and involved seven different residues, which include Asp145 with the lowest distance: 1.829 Å only. A network of hydrophobic bonds further supported the established H-bonds. The identified compounds in honey established one to eight H-bonds, and the highest number of H-bonds was predicted in compounds one and six with 3 twj macromolecules, and compound seven with 1HD2. The diagram of interactions (Figure 5) shows involvement of Asp86, Glu81, Ile10, Leu83, Phe80, Lys33, and Asp145. The latter interacted with a salt bridge bond, which is even stronger than an H-bond. In our study, the number of the closest interacting residues ranged between three and nine. The highest number was predicted in 3TWJ with compound six and in 1DI8 with compounds three, six, seven, nine, and 11. Despite miraxanthin-III (compound eight) interacting with seven residues of 1DI8, it was also deeply embedded in the pocket region of the receptor. In this context, all honey compounds were found to be in close vicinity of all the targeted receptor with a distance of less than three Å. In fact, it exceeded 2.5 only in 3TWJ-compound seven, with a distance equal to 2.556 Å.

## 4. Discussion

With the increasing rates of cancer globally and drug resistance in severe malignancies, finding new, efficient, and safe therapies is a challenge [29,30]. Natural products, such as honey, could be a valuable source of interesting bioactive compounds with prominent anticancer potential [31,33,35,36]. This activity may vary according to floral origin, geographic region, season, or altitudes [4,5]. More than 200 honey constituents have been identified, including sugars, amino acids, polypeptides, minerals, and vitamins. The biological activities of honey are not only due to their high content of phenolic and flavonoid compounds, but also to other active family of compounds, which should be explored [3,37,38].

In our investigation, profiling of ZH via the LC-MS approach reported 11 dominant phytoconstituents belonging to several families, including sugars, amino acid derivatives, alkaloids, and fatty acids. It has been previously reported that the Ziziphus genus, especially *Z. jujuba*, are a source of biological compounds such as alkaloids, glycosides, saponins, flavonoids, terpenoids, and phenolic compounds [39,40]. *Ziziphus* members are known to possess several pharmacological properties, including antioxidant, anticancer, anxiolytic, immunostimulant, antiulcer, anti-inflammatory, cardiovascular, antifertility, contraceptive, antifungal, hypoglycemic, anti-allergic, and anti-diarrheal properties [39]. Additional properties have been reported for some *Ziziphus* members such as the Z. lotus plant species, described to possess the ability to inhibit copper corrosion in natural seawater [41].

Among the identified sugars, isomaltulose constitutes one of the predominant natural carbohydrates found in honey [42]. This sugar is often used as an alternative sweetener with low glycemic index properties and has potential prebiotic benefits [43]. Among the identified alkaloids, anabasamine was previously reported in the seed extract of the *Anabasis aphylla* plant. Due to its acetylcholinesterase inhibitor effect, this alkaloid could be used for symptomatic treatment of dementia [44]. A previous study also reported that ZH could contain protein components, even cyclic peptides, with potential medicinal values [45]. These proteins can be used as a marker for floral origin [46]. In our case, we report a tripeptide Asp-Thr-Gly, which could be a specific marker for the floral origin of our investigated ZH. Among identified compounds, we noted amino acids and amino acid derivatives, such as Miraxanthin-III, N-(1-Deoxy-1- fructosyl) leucine, and N-(1-Deoxy-1- fructosyl) phenylalanine. Miraxanthin-III was previously reported in several extracts issued from medicinal plants, mainly *Ardisia japonica*, *Ligusticum chuanxiong*, *Lippia nodiflora*, and *Mirabilis jalapa* [47], and was classified as an alkaloid within the amine group. Several in silico studies revealed an interesting application for Miraxanthin-III to treat several diseases. It has been shown that this compound could be a prominent alternative for stroke treatment by interacting and activating neuroglobin [48]. Another study provided new insight to use Miraxanthin-III to treat Alzheimer disease by inhibiting proteins that are involved in pathogenesis pathways [47]. This phytocompound could also be used as an alternative treatment for cancer by inhibiting lactate dehydrogenase enzymes leading to decreased cell proliferation and increased sensitivity to chemotherapy agents [49].

Studies revealed that honey may exhibit chemopreventive and therapeutic effects against several cancer forms. Several investigations highlight that honey may display anticancer potential involving different molecular mechanisms, such as cell cycle arrest, apoptosis induction, and activation of mitochondrial pathways, with immunomodulatory and anti-inflammatory effect [3,50,51,52]. ZH has been widely investigated for its healing properties, especially against microbial infectious diseases [53]. Important antioxidant, anti-inflammatory, and antitumor activity has also been reported by several other studies, showing ZH to be an important research topic for alternative treatments of cancer [12]. Through this study, we investigate for the first time the anticancer potential of ZH derived from the Hail region against three cancer cell lines, including breast (MCF-7), lung (A549), and colon (HCT-116). We reported interesting anticancer activity with IC_50_ values in breast 5.203%, followed by colon 6.02%, in favor of inhibition of proliferation. The inhibition was observed in a dose-dependent manner.

Previous studies investigated the anticancer activity of ZH from Egypt against liver Hep-G2, colon Caco-2, and breast MCF-7 cell lines when treated with 100 µg/mL, and revealed a percentage of IC_50_ cytotoxicity of 15.94%, 34.22%, and 34.22%, respectively [54].

The in vitro study showed the chemoprotective effects of ZH by improving breast cancer treatment. They revealed that ZH modulates the expression of the two genes MMPs and TIMPs involved in cancer metastasis in triple-negative breast cancer cells (MDA-MB-231) [18]. This finding may explain the antiproliferative effect observed against the breast MCF-7 cell line in our study. Ghramh and colleagues tested three ZHs from different geographical locations in Saudi Arabia and Pakistan and their synthesized silver nanoparticles (AgNPs). They revealed a growth modulation in normal splenic cells and an antiproliferative effect on Hela cancer cell line HepG2 [55]. Moreover, another study which investigated *Ziziphus* jujube mill honey from China revealed an anticancer effect on HepG2 cells. It showed that this type of honey may block the cell cycle progression at the G0/G1 and induce apoptosis via molecular mechanisms, including DNA damage, p53 expression, and caspase activation [56]. The anticancer potential cannot be related only to phenolic compounds but also to the immunomodulatory effect exhibited by some glycoproteins and glycopeptides [29,57] as previously identified in ZH.

Studies revealed that normal cellular metabolism leads to the accumulation of free radicals, principally reactive oxygen and nitrogen species, that may be enhanced under stressed conditions. This accumulation could give rise to diseases and promote cancers by inducing tumorigenesis processes. Under extreme oxidative stress, the antioxidant agents produced by cells become insufficient [33,36,58]. In this instance, research may turn to the natural antioxidants derived from natural products, especially honey, to overcome oxidative stress and prevent cancers. Studies revealed that honey could be an interesting source of natural antioxidants due to its high content of polyphenols and flavonoids [58,59]. These two compounds add to other molecules, such as vitamins, and some peptides could exhibit chemoprevention activity trough potent free radical scavenging abilities [28,31,57].

In vivo and in vitro assays proved that ZH may improve considerably the oxidative status. Previous studies highlighted that ZH can display in vivo preventive effects against ethanol-induced gastric ulceration in rats by displaying antioxidant and anti-apoptotic effects [16]. Another recent study also demonstrated the gastroprotective effects of Saudi Sidr honey which exhibited high antioxidant activity against acetyl salicylic acid-induced gastric ulcers in rats [8].

An in vivo study showed that the administration of Saudi Sidr honey in rats could prevent the biochemical and histomorphological alteration induced by carbon tetrachloride CCl4 in liver and kidney tissues [60]. All these findings highlight the antioxidant potency that could display the preventative effects of ZH.

Honey content and antioxidant potential may vary according to many factors, including botanical source, region, climatic conditions, seasonal factors, entomological origin, altitude, etc. [61,62]. Thus, this study aimed to investigate the antioxidant potential of identified phytochemicals in ZH derived from the Hail region and to characterize its specificity. Since honey may contain a different group of antioxidants and may display complex oxidation processes [19,60], we used three different approaches based on the electron or hydrogen transfer reaction to assess antioxidant potential: DPPH radical scavenging activity, ABTS radical scavenging activity, and β- carotene-linoleic acid bleaching (BCB) assay. The respective antioxidant potential IC_50_ for these three approaches were, respectively, 3.450 mg/mL, 3.554 mg/mL, and >5 mg/mL.

In a previous study established by Alotibi et al, exploring the biological activity of several Saudi honeys, including unifloral and multiforal Sidr honey, the rate of TPC was between 1.07–1.73 mg/g and the antioxidant activities using DPPH were between 13.56% and 30%. In these studies, there is no clear correlation between activities displayed by tested honey and total phenolic compounds [63,64]. Another study on Omani honey including 10 ZH samples revealed the absence of correlation between TPC, TFC, and antioxidant activity [65]. In that study, the TFC and TPC ranged between 718–1034 mg/kg and 972–1520 mg/kg, respectively, while the antioxidant IC_50_ was estimated between 33.8–72.3 mg/mL using the DPPH approach [58].

In our study, the TPC was 3.396 mg/g and the TFC was 0.061 mg/g, and the obtained antioxidant activity IC_50_ was 3.450 mg/mL using DDPH approaches. Despite the TPC of ZH from the Hail region being lower than Omani ZH, our honey shows higher antioxidant activities. All these findings indicate that the antioxidant activity is not only due to phenolic compounds, but also to other honey constituents that could display antioxidant activity.

We investigated the drug-likeness properties of identified compounds to assess their potential use for drug development. We concluded that the majority of the identified compounds met Lipinsli’s rule. The only compounds that did not meet this rule were still associated with zero alert. Usually, the absence of toxicophores of alarming chemicals indicates the absence of DNS and/or protein damage [30,31,34]. The bioavailability scores showed that all compounds may be physiologically active. Furthermore, BAS scores showed that oral administration is computationally possible (radar figure). Gastrointestinal (GI) and blood-brain-barrier (BBB) were also assessed. Our findings suggest that half of the identified compounds (3, 8–11) have high GI absorption. Most of the compounds were also predicted to be able to penetrate the BBB. This was confirmed by the boiled-egg model (Figure 3). Only four compounds were found to behave as P-glycoprotein (P-gp) substrates. As cytochrome P450 enzymes (CYP) play a key role in drug metabolism and excretion [29,32,34], the major five CYP isoforms (CYP1A2, CYP2C9, CYP2C19, CYP2D6, and CYP3A4) were assessed. While none of the compounds inhibited CYP2C19, CYP2D6 was predicted to be inhibited by three different compounds (three, nine, and 10). Furthermore, compounds one, two, five–eight, and 11 were predicted to inhibit none of the CYP assessed isoforms. Based on the physico-chemical properties, particularly the lipophilicity and molecular weight, skin permeability was evaluated. It ranged between −4.45 and −11.9, which indicated low to moderate permeability [30,32].

The identified compounds in honey were subjected to computational modeling to assess their molecular interactions with some key receptors related to both antioxidant and anticancer activities. Table 4 shows that all compounds bound the three targeted receptors with negative free-binding energy, but with different scores. The free-binding energy ranged between −4.3 and −8.7 kcal/mol. Variation in such score values was reported to be related to the 3D chemical structures of the ligands [30,34,66]. The best binding score was predicted in the 1di8- Miraxanthin- III (compound eight) complex. Miraxanthin-III was also found to interact with the 3twj receptor with −8.0 kcal/mol. The molecular interactions of this compound with 1di8 included four conventional H-bonds and involved seven different residues, which include Asp145 with the lowest distance: 1.829 Å only. Regardless of H-bonds that are commonly evaluated to assess the biological activities of the targeted compounds [29,31,32], a network of hydrophobic bonds was also found within the different studied complexes. This may contribute to the stability of the complexes as reported in several recent in silico studies [29,30,67,68]. Our results exhibit that all identified compounds in honey established one to eight H-bonds. The highest number of H-bonds (n = 8) was predicted in compounds one and six with 3twj macromolecules and compound seven with 1hd2. The corresponding diagram of interactions (Figure 3) showed involvement of Asp86, Glu81, Ile10, Leu83, Phe80, Lys33, and Asp145. The latter interacted with a salt bridge bond, which is even stronger than an H-bond. Interactions with key residues were found to promote biological activities, including antioxidant and anticancer potential in the studied compounds. In our study, the number of the closest interacting residues ranged between three and nine. The highest number was predicted in 3twj with compound six and in 1di8 with compounds three, six, seven, nine, and 11. As CDK-2 and ROCK 1 are involved in the control of cell cycles and are commonly targeted to counteract cancer cell proliferation [30,66], our data may confirm the potential anti-tumoral effect of ZH phytochemicals. Despite miraxanthin-III (compound eight) interacting with seven residues of 1di8, it was also deeply embedded in the pocket region of the receptor. In this context, all honey compounds were found to be in close vicinity of all the targeted receptor with a distance of less than three Å. In fact, it exceeded 2.5 only in 3twj-compound seven with a distance equal to 2.556 Å. Ligand deep embedding was reported to enhance biological activity via increasing the complex stability [30,33,68]. Taken together, these molecular interactions justify that the antioxidant and anticancer potentials of the studied honey are thermodynamically possible, and this could explain the results obtained in vitro.

## 5. Conclusions

The diversity and complexity of honey phytocompounds and the absence of standardization of its biological activities pose a hindrance to use honey in conventional medicine. For that, extended studies are needed to characterize honey constituents and explore their healing properties for use in alternative medicines. Through our findings, we reported interesting antioxidant activity that could be related to non-phenolic or flavonoid compounds. The ZH also showed a notable cytotoxic effect in a dose-dependent manner against three cancer cell lines in favor of anticancer potential. The computational analysis revealed that most honey compounds present good drug-likeness properties with imminent antioxidant and anticancer effects, mainly for L-Gulonate, Isomaltulose, Semilepidinoside B, and Miraxanthin-III. These findings may support the in vitro observed results. Nonetheless, further studies on the isolation, characterization, and assessment of these biological activities should be conducted to support the above-stated findings. The findings of our investigation proves that ZH could be used for therapeutic purposes to treat cancers.

## Figures and Tables

**Table 1:** Phytochemical compounds identified by the HR-LCMS technique in *Ziziphus* honey from the Hail region.

N°	Compound Name	Family	RT (mn)	MW (g/mol)	Chemical Formula	[*m/z*]−	[*m/z*]+
**1**	L-Gulonate	Sugar Acid	1.08	196.0576	C_6_ H_12_ O_7_	195.0503	-
**2**	N-(1-Deoxy-1-fructosyl) leucine	Amino acid derivative	1.238	293.147	C_12_ H_23_ N O_7_	-	294.1543
**3**	Anabasamine	Alkaloids	1.389	253.1545	C_16_ H_19_ N_3_	-	276.1435
**4**	N-(1-Deoxy-1- fructosyl) phenylalanine	Amino acid derivative	1.672	327.1307	C_15_ H_21_ N O_7_	-	328.138
**5**	Asp-Thr-Gly	Tripeptide	2.082	291.1107	C_10_ H_17_ N_3_ O_7_	-	292.1178
**6**	Isomaltulose	Glucans	2.503	342.1158	C_12_ H_22_ O_11_	-	365.104
**7**	Semilepidinoside B	Glycoside	2.734	366.1415	C_17_ H_22_ N2 O_7_	-	367.1491
**8**	Miraxanthin-III	Amino acid derivative	3.67	330.1215	C_17_ H_18_ N_2_ O_5_	-	331.1284
**9**	Anatalline	Alkaloid	5.935	239.1418	C_15_ H_17_ N_3_	-	240.1492
**10**	7-hydroxy-10E,16-heptadecadien-8-ynoic acid	Fatty acid	20.175	278.1936	C_17_ H_26_ O_3_	277.1866	-
**11**	14-fluoro-myristic acid	Fatty Acyl	27.174	246.2024	C_14_ H_27_ F O_2_	291.2009	

RT: Retention time (mn); MW: molecular weight (g/mol); [*m*/*z*]−: mass-to-charge ratio in negative ionization mode; [*m*/*z*]+: mass-to-charge ratio in positive ionization mode.

**Table 2:** Antioxidant activities of the ZH sample compared with butylated hydroxytoluene (BHT) and ascorbic acid (AA).

	*Ziziphus* Honey	(BHT)	(AA)
**Phytochemical Classes**			
Total Flavonoids Content (mg QE/g)	0.061 ± 0.001 ^A^	-	-
Total Tannins Content (mg TAE/g)	2.511 ± 0.321 ^B^	-	-
Total Phenols Content (mg GAE/g)	3.396 ± 0.019 ^C^	-	-
**Antioxidant tests**			
DPPH IC_50_ (mg/mL)	3.450 ± 0.081 ^b^	0.023 ± 3 × 10^−4a^	0.022 ± 5 × 10^−4a^
ABTS IC_50_ (mg/mL)	3.554 ± 0.139 ^a^	0.018 ± 4 × 10^−4a^	0.021 ± 0.001 ^a^
β-carotene IC_50_ (mg/mL)	5 ^c^	0.042 ± 3.5 × 10^−3b^	0.017 ± 0.001 ^a^

The letters (a–c) indicate a significant difference between *Ziziphus* honey results by using the different antioxidant tests compared with standard and drugs according to the Duncan test (*p* < 0.05). Capital letters (A–C) indicate a significant difference between phytochemical method screening using the Duncan test (*p* < 0.05).

**Table 3:** Drug-likeness, pharmacokinetics, and physico-chemical properties of the identified compounds in honey (**1**–**11**) based on absorption, distribution, metabolism, elimination, and toxicity (ADMET) properties.

Entry	Honey Compounds										
	1	2	3	4	5	6	7	8	9	10	11
Properties/Lipophilicity/Drug-likeness											
Molecular weight	195.15	293.31	253.34	327.33	291.26	342.30	366.37	330.34	239.32	278.39	246.36
Num. heavy atoms	13	20	19	23	20	23	26	24	18	20	17
Num. arom. heavy atoms	0	0	12	6	0	0	11	6	12	0	0
Fraction Csp3	0.83	0.92	0.38	0.53	0.60	1.00	0.47	0.24	0.33	0.59	0.93
Num. rotatable bonds	5	7	2	7	10	5	6	6	2	11	13
Num. H-bond acceptors	7	8	3	8	8	11	8	6	3	3	3
Num. H-bond donors	5	6	0	6	6	8	5	4	1	2	1
Molar Refractivity	36.59	68.03	81.17	78.09	63.60	68.16	88.68	92.38	75.32	83.98	71.23
TPSA (Å^2^)	141.28	139.48	29.02	139.48	179.05	189.53	137.29	119.22	37.81	57.53	37.30
Consensus Log Po/w	−2.87	−1.92	2.33	−1.52	−2.66	−3.49	−0.33	0.75	1.94	3.70	4.25
Lipinski’s Rule	Yes	Yes	Yes	Yes	Yes	No	Yes	Yes	Yes	Yes	Yes
Bioavailability Score	0.56	0.55	0.55	0.55	0.11	0.17	0.55	0.56	0.55	0.85	0.85
PAINS	0 alert	0 alert	0 alert	0 alert	0 alert	0 alert	0 alert	0 alert	0 alert	0 alert	0 alert
Pharmacokinetics											
GI absorption	Low	Low	High	Low	Low	Low	Low	High	High	High	High
BBB permeant	No	No	Yes	No	No	No	No	No	Yes	Yes	Yes
P-gp substrate	No	Yes	Yes	No	No	Yes	No	No	Yes	No	No
CYP1A2 inhibitor	No	No	No	Yes	No	No	No	No	No	Yes	No
CYP2C19 inhibitor	No	No	No	No	No	No	No	No	No	No	No
CYP2C9 inhibitor	No	No	No	No	No	No	No	No	No	Yes	No
CYP2D6 inhibitor	No	No	Yes	No	No	No	No	No	Yes	Yes	No
CYP3A4 inhibitor	No	No	Yes	No	No	No	No	No	No	No	No
Log Kp (cm/s)	−9.88	−10.21	−6.74	−10.24	−11.90	−11.41	−9.20	−7.32	−6.75	−5.31	−4.45

**Table 4:** Free binding energy and molecular interactions of the identified compounds in honey with the targeted receptors: 1jij, 1hd2, 1di8, and 3twj.

N°	Target Receptor	Binding Energy (kcal/mol)	Molecular Interactions			
			No. H-Bonds	No. Closest Interacting Residues	Closest Interacting Residue	Distance (Å)
**1**	1HD2	−5.0	6	7	Val94	2.003
	1DI8	−5.0	6	6	Lys33	1.946
	3TWJ	−5.7	8	8	Lys200	1.751
**2**	1HD2	−5.2	7	7	Ala42	1.888
	1DI8	−6.5	3	8	Asn132	2.064
	3TWJ	−6.3	5	6	Gly85	1.879
**3**	1HD2	−6.0	1	3	Lys22	2.193
	1DI8	−8.4	2	9	Gln131	1.881
	3TWJ	−7.2	2	6	Ala234	2.385
**4**	1HD2	−6.6	7	6	Val75	2.064
	1DI8	−7.9	6	8	Asn132	2.001
	3TWJ	−7.5	6	5	Lys200	1.852
**5**	1HD2	−5.5	6	6	Val94	2.351
	1DI8	−6.3	6	4	Asp145	2.155
	3TWJ	−6.8	7	7	Lys200	1.889
**6**	1HD2	−6.0	6	8	Gly82	2.081
	1DI8	−6.6	5	5	Glu12	1.908
	3TWJ	−7.0	8	9	Ala234	2.238
**7**	1HD2	−6.3	8	7	Asn21	2.093
	1DI8	−8.0	5	9	His84	1.994
	3TWJ	−7.7	4	8	Cys220	2.556
**8**	1HD2	−6.2	5	6	Arg95	2.095
	1DI8	−8.7	4	7	Asp145	1.829
	3TWJ	−8.0	6	7	Ala119	1.943
**9**	1HD2	−6.1	4	4	Asp7	2.036
	1DI8	−8.3	3	9	Asp145	2.389
	3TWJ	−7.7	3	5	Glu24	2.019
**10**	1HD2	−5.1	4	5	Leu96	2.118
	1DI8	−6.7	3	8	Leu83	1.865
	3TWJ	−5.3	5	6	Ala234	1.976
**11**	1HD2	−4.3	4	5	Leu96	2.204
	1DI8	−6.0	1	9	Lys33	2.151
	3TWJ	−5.5	3	7	Thr219	2.092

## Data Availability

Not applicable.

## Supplementary Materials

The following supporting information can be downloaded at: https://www.mdpi.com/article/10.3390/life13010035/s1, Figure S1: HR-LC/MS spectrum peak of honey showing the chromatogram intensity against the acquisition time, (A) positive analysis and (B) negative analysis.
