# Supplementary material for: Phytochemical Composition, Antioxidant, and Anticancer Activities of Sidr Honey: In Vitro and In Silico Computational Investigation
Source: Life (Basel). 2022 Dec 23;13(1):35. doi: 10.3390/life13010035 (PMC9867352; doi:10.3390/life13010035)
Supplement: Supplementary file 1 [file life-13-00035-s001.zip › life-2088809-supplementary.pdf]

Supplementary Materials

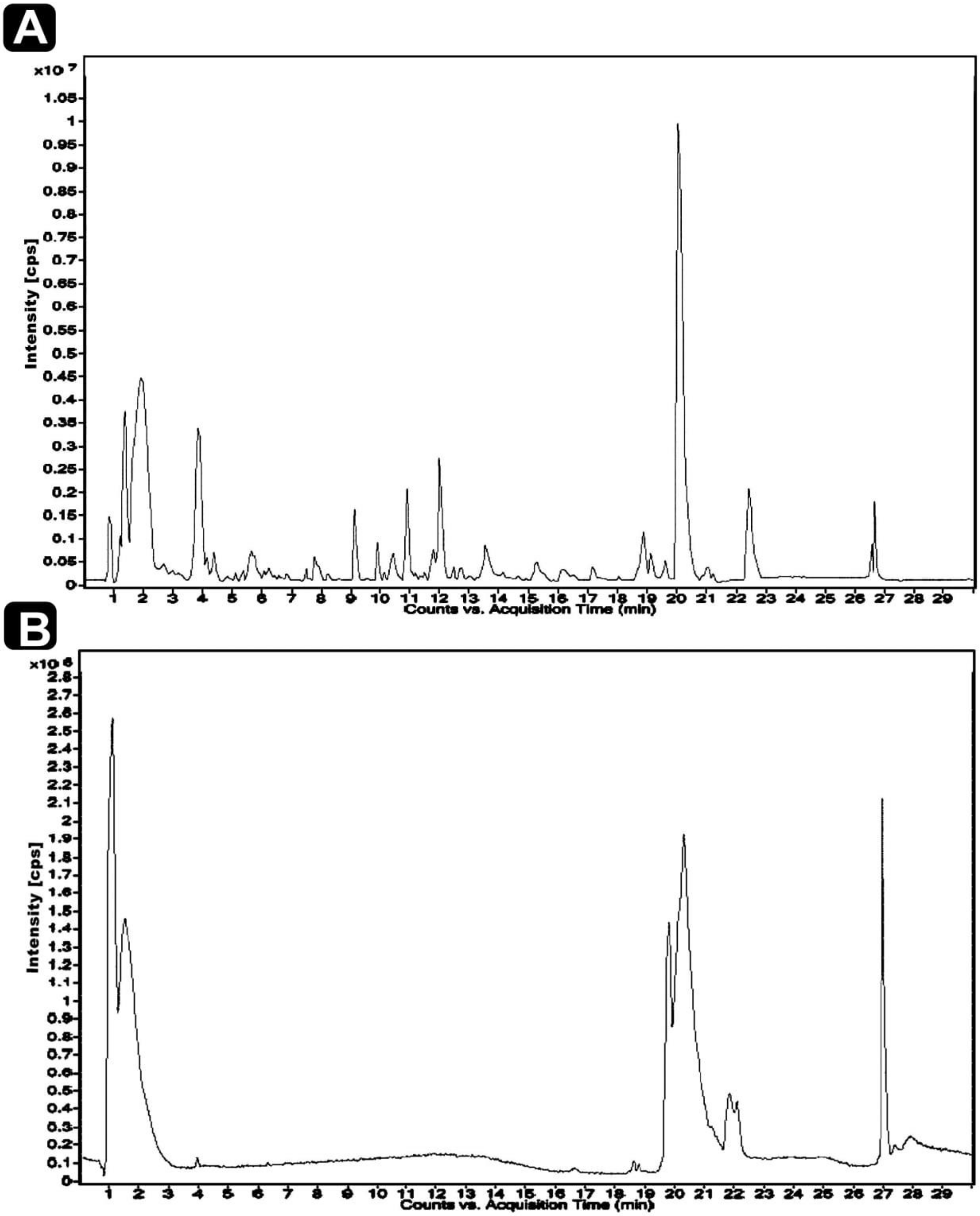

Figure S1. HR-LC/MS spectrum peak of honey showing the chromatogram intensity against the acquisition time, (A) positive analysis and (B) negative analysis.
